# Cultivating competence in error management: The development and impact of a tailored quality improvement and patient safety curriculum in pathology training

**DOI:** 10.1016/j.acpath.2026.100245

**Published:** 2026-03-19

**Authors:** Nikka Khorsandi, Kara Tanaka, Elena Nedelcu, Sarah Calkins, Kristie White

**Affiliations:** a Departments of Pathology and Laboratory Medicine, University of California San Francisco, San Francisco, CA, USA; b Peninsula Pathologists Medical Group, South San Francisco, CA, USA

**Keywords:** Curriculum development, Diagnostic error, Pathology education, Patient safety

## Abstract

Despite recent efforts in medicine to minimize diagnostic errors in healthcare, a gap persists in the formal education of pathology trainees in quality improvement and patient safety and error management. This study evaluates a formal curriculum developed and implemented over one year at a pathology residency training program aimed at addressing this educational gap for pathology trainees. A year-long curriculum was developed and implemented involving didactics, small group discussions, and case-based exercises known as error management cases of the week. Pre- and post-curricular surveys assessed trainees' attitudes, beliefs, skills, and confidence related to quality improvement and patient safety. The curriculum's success was measured via participation rates, changes in trainees' attitudes, beliefs, skills, and confidence related to quality improvement and patient safety as measured through surveys, and longitudinal comparisons with national resident survey results. The pre-survey revealed a pressing need for quality improvement and patient safety training, especially among early trainees. Postcurriculum, trainee participants demonstrated significant improvement in confidence and application of quality improvement and patient safety methodologies. This was further supported by an increase in the pathology trainees’ participation in safety event investigations compared to averages from a national survey. The study highlights the effectiveness of a structured quality improvement and patient safety curriculum in enhancing trainee competence and confidence, suggesting a template for wider adoption in pathology residencies. The curriculum's success in shifting attitudes and skills advocates for its integration into national residency programs, promoting a culture of safety and open error management in the field of pathology.

## Introduction

The landmark Institute of Medicine (IOM) report *To Err is Human,* brought recognition to both the frequency and grave importance of patient safety and error management in the US healthcare system.^1^ With this highlighted focus on medical errors, the field of patient safety and quality improvement underwent significant expansion.^2^ For diagnostic specialties such as pathology and laboratory medicine, this call to action is further described in a subsequent report, *Improving Diagnosis in Health Care*.^3^ This report focused on diagnostic errors in medicine and highlighted the ubiquity with which diagnostic errors occur and the importance of learning from prior diagnostic errors and near misses.^3^^,^^4^ Specific recommendations in pathology arising from this report included creating a system and culture that supports improvements in diagnostic performance.^4^

Since then, the fields of anatomic and surgical pathology have implemented various strategies to improve patient safety including second pathologist review of cases to identify disagreements and interpretive errors, pathologists assisting with interpretation of pathology reports in multidisciplinary meetings, and implementation of systematic synoptic reporting in pathology reports.^5^, ^6^, ^7^, ^8^, ^9^, ^10^ While each of these strategies has been shown to improve patient safety, reported barriers to improving patient safety still exist including embarrassment from making errors and lack of confidence in an individual's own error disclosure skills.^6^^,^^10^^,^^11^

This lack of confidence in error disclosure is highlighted in a survey of pathologists in which over 75% recognized the gravity of medical errors and almost all respondents had been involved with an error; however, a much smaller proportion had experience disclosing major errors to patients (16.2%).^12^ One contributor to this obstacle stems from conflicting guidance from risk managers advocating that physicians should never apologize to prevent potential litigation.^13^ Regardless, there is a broad and growing movement for pathologists to actively communicate with patients about potential medical errors.^14^

To begin preparing future pathologists in patient safety and error management, the Accreditation Council for Graduate Medical Education (ACGME) has introduced a milestone for all US Pathology residents which deems the expert resident (a Level 5 rating) should be able to create quality improvement (QI) initiatives, engage with processes to prevent patient safety events, and mentor others in patient safety event disclosure.^15^ However, formal, published pathology residency curricula in this area are limited.^16^, ^17^, ^18^ This need for greater engagement with pathology trainees is highlighted both in publications^19^^,^^20^ and by recent ACGME resident surveys, which show that pathology trainees nationally rate their training in the areas of “participating in safety event investigations and analyses” well below the average compliance rating for all medical subspecialties surveyed. In addition, our program average was well below the specialty mean in this area.

To expand the limited number of strategies for pathology resident education in quality improvement and patient safety (QIPS), the following curriculum was developed, implemented over one year, and evaluated based on trainee confidence in QIPS topics.

## Materials and methods

### Setting

This study was performed within a residency program within the Departments of Pathology and Laboratory Medicine of a large tertiary care center that trains 45–50 residents and fellows each year (collectively referred to as “trainees”). The program offers residency training tracks in anatomic pathology, clinical pathology, and neuropathology in addition to numerous general and subspecialty fellowships across five campuses.

### Curriculum

**Fig. 1:**
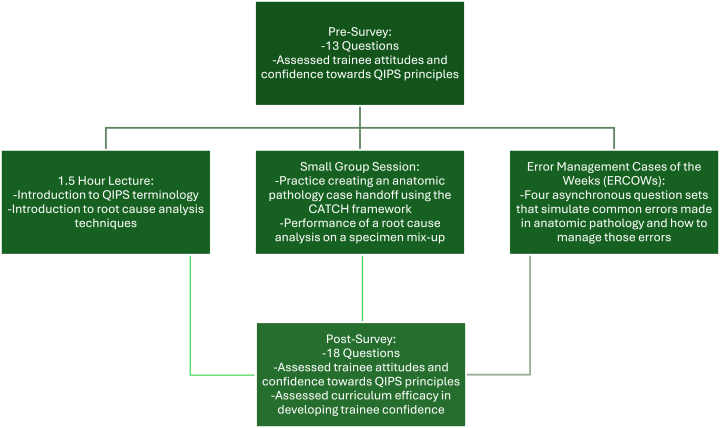
Outline of QIPS-dedicated curriculum and assessment surveys. QIPS: quality improvement and patient safety.

There is a paucity of pathology trainee-specific QIPS curricula in the literature. To alleviate this gap, we utilized Kern's six-step approach to medical education curriculum development^21^ to build a year-long curriculum with the goals of increasing trainees' exposure to QIPS principles, and to provide practice in the application of QIPS methodology to clinical problem solving. Based on Kern's six-step development approach, the problem was identified that the current literature recognizes the limited formal QIPS training in pathology residency. A targeted needs assessment involved residents self-rating that their training in formal QIPS was lacking and below the national average. An overall objective to improve resident confidence in basic QIPS topics was set for the academic year as assessed by trainee responses to both a modified anonymous survey and the ACGME residents' surveys. More specific objectives provided to trainees covered cognitive, affective, and psychomotor domains. Some of these specific objectives included: “Understanding and describing the elements of a comprehensive and concise handoff between trainees for communicating outstanding surgical cases”, “evaluate a (Clinical summary, Active issues, Tests, Contingency plan, Hear it back (CATCH)-handoff from a co-resident and provide constructive feedback”, and “learn from the root causes and apply that information to make systems-level changes to prevent future specimen mix-ups”. The curriculum was designed to include a multitude of educational strategies including a lecture on QIPS principles and methodologies, a small-group session to practice performing a root cause analysis and a patient handoff, a series of asynchronous case-of-the-week question sets aimed at managing errors, and pre-and post-surveys to assess respondent attitudes, beliefs, skills, and confidence towards QIPS. The curriculum was designed and created by two residents (NK and KT) and three faculty members (KW, EN, SC) for implementation during the 2021–2022 academic year [Fig. 1]. Evaluation and feedback were garnered with a post-curriculum implementation survey in which data were compared to pre-survey data. In addition, data from the ACGME residents' surveys were compared from before and after implementation of the curriculum to assess for impact.

### Lecture and small group

**Fig. 2:**
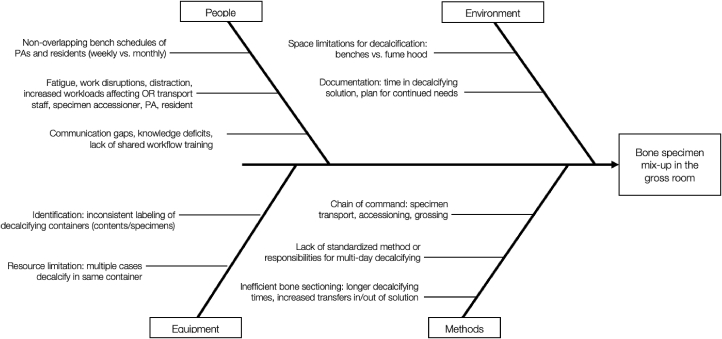
An example of fishbone diagram root cause analysis (RCA) to solve a gross room specimen mix-up error. PA: pathologists’ assistant; OR: operating room.

**Fig. 3:**
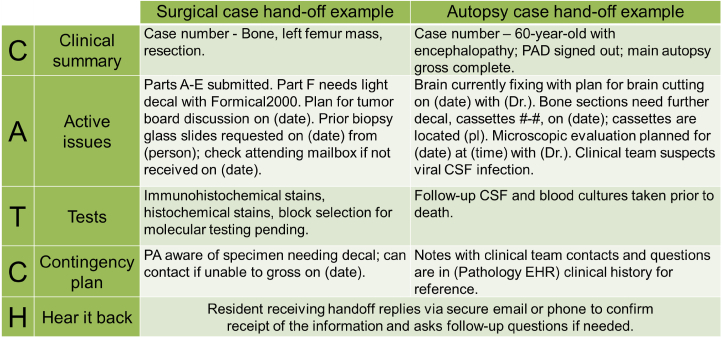
Examples of CATCH framework utilization for end-of-rotation clinical hand-offs to improve communication and optimize patient care. PA: pathologists' assistant; PAD: preliminary autopsy diagnoses; CSF: cerebrospinal fluid; EHR: electronic health record; pl: place.

Two faculty members (EN, KW) delivered a 90-min lecture on basic QIPS principles and methodologies during the weekly departmental grand-rounds. Trainees then engaged in a 90-min flipped-classroom small group (SG) session, conducted over Zoom (Zoom Video Communications, San Jose, CA, USA) to allow participation at all clinical sites. The SG revolved around clinical utilization of QIPS tools applied to case-based anatomic pathology scenarios: trainees used root cause analysis (RCA) using a fishbone diagram^22^^,^^23^ to solve a gross room specimen mix-up error and used the pathology-specific CATCH framework^24^ for end-of-rotation clinical hand-offs to improve communication and optimize patient care [Fig. 2, Fig. 3]. Related literature was provided as asynchronous pre-session reading.

Trainees received an introductory QIPS lecture led by an Institute for Healthcare Improvement-certified QIPS expert and then divided into multiple break-out groups to work through the two cases. Each break-out group was led by trainee facilitators who received additional instruction on how to guide their group through the cases and supplemental materials to provide if requested by the participants. Groups sent their responses to the case questions to the principal facilitators, which were reviewed collectively in a large group discussion. Participants had an opportunity to discuss approaches to error identification and management including information gathering, systems analysis, and environmental and human contributors to error.

### Error management cases of the week

Following the lecture and SG session, four asynchronous problem sets termed “error management cases of the week (ERCOWs)” were sent to trainees. Prior to being sent to trainees, the ERCOWs were reviewed by two attending pathologists (KW, SC) to ensure accuracy, relevance, and alignment of the ERCOW cases with the curriculum's learning objectives. The ERCOWs were sent to trainees monthly for four months total. The ERCOWs followed a familiar surgical pathology unknown case-of-the-week format used by the institution, with a specialized focus on identifying and managing errors in anatomic pathology. Question sets were designed to have trainees experience making a diagnostic error and the subsequent management steps necessary to rectify the situation. These question sets were based on prior real-life errors within the department, which were modified and anonymized. These cases were identified by querying the meeting minutes for the departmental quality improvement committee’s quarterly meeting minutes. At these meetings, any quality and safety issues such as diagnostic discrepancies and report amendments are reviewed and discussed. By using these cases and anonymizing the details of the case, our goal was to highlight to the trainees that errors will inevitably occur at some point during one's career and understanding how to manage these errors is vastly important.

In addition, one board-style question and one lab management question were included in each ERCOW and were adapted from questions in various laboratory management resources purchased by the program or freely available to the trainees.^25^^,^^26^ The ERCOWs were sent to all trainees within the program. Anonymous responses were collected for two weeks via the Qualtrics XM electronic survey tool (Qualtrics International Inc., Provo, UT, USA). Responses were reviewed to identify any areas requiring significant clarification, the answers and explanations were sent to all trainees, highlighting the learning points from each case. Each question set also contained an introductory message highlighting the fact that each of the cases was modified from prior errors made within the department to encourage future learning from past errors. A total of four question sets were sent throughout the curriculum's implementation. An example question set of the ERCOW can be found in Supplemental Material 1. Additionally, other aspects of the curriculum can be provided upon reasonable request.

### Pre- and post-survey

Prior to initiation of the curricular components, all trainees were asked to participate in an anonymous 13-question “presurvey” delivered via Qualtrics. The survey was modified from a previously published survey assessing beliefs, attitudes, skills, and confidence of medical students toward quality improvement.^27^ Our modified survey focused on the importance of QIPS in the context of pathology. Survey questions utilized a 5-point Likert scale to assess respondents’ level of agreement or disagreement with statements on beliefs about QIPS topics and confidence in implementing QIPS topics (1 = strongly disagree, 2 = somewhat disagree, 3 = neither agree nor disagree, 4 = somewhat agree, 5 = strongly agree). This survey collected limited demographic data (postgraduate year (PGY), prior experience with QIPS topics) to provide subgroup analysis while ensuring anonymity of responses. The survey was sent via email from the program director to all 46 residents and fellows at the training program with responses collected via the electronic survey tool Qualtrics (Provo, UT). Survey responses were collected at the start of the academic year and prior to implementation of any curriculum components.

At the completion of the year-long curriculum, trainees participated in an anonymous 18-question “post-survey”, containing the same 13 questions as the presurvey with 5 additional questions related to participation in the QIPS curriculum. Trainees were asked if they participated in the lecture, SG, and/or the ERCOWs. If they did participate, they were asked if the activity was helpful in learning QIPS topics. Finally, qualitative feedback regarding the curriculum was solicited in an open-ended free-text format.

### Data analysis

Statistical analyses were conducted with R Statistical Software (v4.1.2; R Core Team 2021) with descriptive statistics characterizing responses to each question. Respondents to the presurvey were assessed according to two distinct parameters: years of training and prior experience with QIPS. The respondents were classified as early training (PGY1-2) or as advanced training (PGY3+), and, separately, into those who reported a history of training in QIPS topics and those without prior experience with QIPS. A student's unpaired *t*-test was used to identify any significant differences between the PGY1-2 versus PGY3+ groups as well as between the respondents with or without a history of training. Prior to each *t*-test, a test to evaluate variance was performed and appropriately applied. Significance was defined as a probability of less than 0.05.

The lecture and SG were assessed by the rate of participation of trainees in the interactive, educational activity. Similarly, assessment of the ERCOWs involved calculating a response rate for each ERCOW set as well as an overall accuracy rate of all participants, participants in early training, and participants in advanced training.

The postsurvey results were divided into respondents who did not participate in the curriculum and those who did participate in the curriculum (participation was defined as engaging in both the lecture/small-group session and by responding to at least one of the ERCOW sets). Individuals who did not participate served as a control group to account for the change in trainee attitudes, beliefs, skills, and/or confidence related to QIPS topics over one year of residency training without engagement with this curriculum. A student's unpaired *t*-test was applied to evaluate for changes in survey scores in the control group as compared to the overall pretest scores. Analysis was also performed to compare survey responses for the trainee group that did participate in the curriculum as compared to the overall pretest scores. Variance evaluation was appropriately applied prior to *t*-test analyses and significance defined as a probability of less than 0.05. The study was deemed exempt per the campus Institutional Review Board.

## Results

The pre-survey garnered 32 trainee responses (response rate 32/46, 70%). All respondents indicated that they felt strongest about understanding that QIPS methods can change a system, the role QIPS plays in the practice of pathology and valuing QIPS training as part of pathology training. However, more respondents disagreed with the statement that they had applied QIPS methods during residency training. When comparing between PGY1-2 and PGY3+, the only difference identified was that PGY1-2 respondents agreed less that they had applied QIPS methods during residency to date as compared to PGY3+ respondents. When comparing between respondents with a history of QIPS training and those without a history of training, respondents with a history of QIPS training had a higher rate of agreement with statements indicating they were more confident in their ability to identify quality gaps, understand root causes of errors, use root cause analysis tools, and design interventions or changes (Table 1).

**Table 1:** Presurvey results assessing pathology trainee attitudes, beliefs, skills and confidence toward quality improvement and patient safety (QIPS).

Question	All respondents (n = 32); mean	Respondent year of training: PGY 1 or 2 (n = 14); mean	Respondent year of training: PGY3+ (n = 18); mean	*P*-value^a^	Respondents with no history of training (n = 18); mean	Respondents with history of training (n = 14); mean	*P*-value^b^
I am interested in QIPS	3.75	3.78	3.72	0.85	3.61	3.93	0.34
I understand the role QIPS plays in the practice of pathology	4.06	3.86	4.22	0.31	3.89	4.29	0.20
I value QIPS training as part of pathology residency training	4.00	3.79	4.17	0.22	4.00	4.00	1.00
Applications of QIPS methodologies can help change a system	4.28	4.29	4.28	0.98	4.28	4.29	0.98
I have applied QIPS methodologies during residency training	2.88	2.29	3.33	0.01^‡^	2.94	2.79	0.73
I feel confident in my ability to understand quality issues	3.81	3.64	3.94	0.37	3.61	4.07	0.17
I feel confident in my ability to identify quality gaps	3.72	3.71	3.72	0.98	3.28	4.29	0.01^c^
I feel confident in my ability to understand root causes of errors	3.81	3.79	3.83	0.90	3.39	4.36	0.004^c^
I feel confident in my ability to apply evidence and best practices of QIPS in pathology	3.13	3.07	3.17	0.74	3.00	3.29	0.32
I feel confident in my ability to use root cause analysis tools in error management	3.19	3.29	3.11	0.65	2.61	3.93	0.0001^c^
I feel confidence in my ability to design an intervention or change	3.03	2.93	3.11	0.61	2.61	3.57	0.005^‡^

Scale: 1 = strongly disagree, 2 = somewhat disagree, 3 = neither agree nor disagree, 4 = somewhat agree, 5 = strongly agree.

a *P*-value of student's t-test comparing PGY1 or 2 respondents to PGY3+ respondents.

b *P*-value of student's t-test comparing respondents with no history of QIPS training to respondents with a history of QIPS training.

c Indicates significance of <0.05. QIPS: quality improvement and patient safety.

Forty-six trainees were invited to participate in the flipped-classroom lecture and SG session. Twenty-six of the trainees completed the lecture and SG session (participation rate of 57%). Response rates to the various ERCOWs ranged anywhere from 26% to 39%. Each set contained between four and nine questions challenging respondents to employ error management skills in mock diagnostic errors. Types of cases covered in the ERCOWs are listed in Table 2; they include cytology-histology discrepancies, reporting urgent values, and frozen section-final histology discrepancies. Finally, a root cause analysis exercise was conducted related to a specimen mix-up in the gross room requiring molecular identity testing. Respondent accuracy to ERCOW sets ranged from 77% to 92.5% (Table 2). Variation in the overall accuracy on the ERCOW questions was largely driven by respondents' knowledge to the individual board review or lab management questions. For example, in the first ERCOW set, respondents' accuracy on the board-style review question was 89%, whereas in the third ERCOW set, respondents’ accuracy on the board-style review question was 23%.

**Table 2:** Overview of ERCOW sets and accuracy of trainee responses.

ERCOW set and response rate	Number of error management questions	Topics covered	Accuracy of PGY1 or 2 respondents	Accuracy of PGY3+ respondents	Overall accuracy of all respondents
ERCOW #1 18/46 (39%)	4 questions	Cyto-histo discrepancy	92.5% (n = 10)	94% (n = 8)	93% (n = 18)
		Frozen-final discrepancy			
		Board style review question			
		Lab management question			
ERCOW #2 12/46 (26%)	4 questions	Catching a colleague's error	82% (n = 7)	75% (n = 5)	84% (n = 12)
		Urgent value in anatomic pathology			
		Board style review question			
		Lab management question			
ERCOW #3 13/46 (28%)	5 questions	Personal misdiagnosis and error management	77% (n = 7)	73% (n = 6)	75% (n = 13)
		Frozen-final discrepancy			
		Board style review question			
		Lab management question			
ERCOW #4 13/46 (28%)	9 questions	Identification, management, and molecular identity testing for a specimen mix-up, sample root cause analysis (fishbone diagram)	92% (n = 7)	94% (n = 6)	93% (n = 13)

ERCO: error management cases of the week.

Twenty-seven trainees responded to the postsurvey (response rate 27/46, 59%). Fourteen of twenty-seven indicated they had participated in the curriculum (52%), whereas the remaining thirteen respondents indicated they had not participated in the curriculum (48%). No differences were identified in survey results between respondents who did not participate in the curriculum and the presurvey results. To evaluate the utility of the curriculum in impacting beliefs, attitudes, skills, and confidence in QIPS, survey data were compared between trainee respondents who participated in the curriculum “postsurvey participants” versus all trainee respondents prior to the start of the curriculum “presurvey,” as well as between trainee respondents who did not participate in the curriculum “postsurvey controls” versus all trainee respondents prior to the start of the curriculum (“presurvey”) (Table 3). Based on the analysis, in respondents who did participate in the curriculum, agreement with the following statements was significantly higher as compared to the presurvey results: “I value QIPS training as part of pathology residency training”, “I have applied QIPS methodologies during residency training”, “I feel confident in my ability to apply evidence and best practice of QIPS in pathology”, “I feel confident in my ability to use root cause analysis tools in error management”, and “I feel confident in my ability to design an intervention or change”. However, when comparing trainees who did not participate in the curriculum to the presurvey results, no statistically significant changes were identified, suggesting involvement with the curriculum improved trainees’ confidence and abilities in the categories listed above.

**Table 3:** Comparison of trainee responses to pre- and post-surveys.

Question	Presurvey (n = 32) Mean	Postsurvey participants (n = 14) Mean, *P*-value^a^	Postsurvey vontrols (n = 13) Mean, *P*-value^a^
I am interested in QIPS	3.75	4.00, 0.36	3.46, 0.34
I understand the role QIPS plays in the practice of pathology	4.06	4.43, 0.09	4.31, 0.41
I value QIPS training as part of pathology residency training	4.00	4.64, <0.01^b^	4.15, 0.58
Applications of QIPS methodologies can help change a system	4.28	4.50, 0.29	4.23, 0.87
I have applied QIPS methodologies during residency training	2.88	3.71, 0.04^b^	3.54, 0.08
I feel confident in my ability to understand quality issues	3.81	4.00, 0.51	4.08, 0.37
I feel confident in my ability to identify quality gaps	3.72	4.07, 0.27	4.08, 0.28
I feel confident in my ability to understand root causes of errors	3.81	4.07, 0.40	4.08, 0.41
I feel confident in my ability to apply evidence and best practices of QIPS in pathology	3.13	3.86, <0.01^b^	3.62, 0.08
I feel confident in my ability to use root cause analysis tools in error management	3.19	4.00, <0.01^b^	3.31, 0.73
I feel confident in my ability to design an intervention or change	3.03	3.86, 0.01^b^	3.46, 0.20

Scale: 1 = strongly disagree, 2 = somewhat disagree, 3 = neither agree nor disagree, 4 = somewhat agree, 5 = strongly agree.

a *P*-value calculated from student's t-test as compared to the pre-survey results.

b Indicates statistical significance. QIPS: quality improvement and patient safety.

Furthermore, review of the pathology residency program's trainee responses to the ACGME resident/fellow survey identified that in 2023, 84% of the program's pathology trainees indicated they had participated in safety event investigation and analysis at some point over the prior academic year. This was an increase from 48% in the prior year. This increase may represent trainees recognizing that participation in this curriculum acted as engagement with safety event investigation and analysis or may represent better awareness of other activities trainees engaged in throughout the year that constitute engagement with safety event investigation and analysis. Additionally, this level of trainee participation in 2023 was higher than the overall pathology compliance with this statement (68%) and the national compliance of all medical residents with this statement (79 %).

## Discussion

These results highlight both the necessity of interventions for QIPS training in pathology training as well as the benefit this curriculum has in improving pathology resident value and confidence in applying QIPS methodologies. While the results from the presurvey indicate that more advanced residents felt they had more experience with QIPS methodologies as part of residency training when compared to early residents, even the agreement with this statement on the part of advanced residents was much lower than anticipated (mean of 3.33 on a scale of 1–5). The highlighting that there was no difference between the presurvey results and the postsurvey results in residents who did not participate in the curriculum, indicates that additional efforts need to be made to integrate QIPS curriculum into residency training. This is further supported by the fact that, when compared to presurvey results, postsurvey respondents who had participated in the curriculum felt significantly more agreement that they had applied QIPS methodologies during residency. This improvement in trainee QIPS application awareness is reflected in other residency subspecialties that indicate that the presence of any QI curriculum improves knowledge of QI^28^, ^29^, ^30^ as well as the improvement reported in the program's ACGME resident survey results. Review of the literature, however, only identifies a few publications related to QIPS curricula in pathology residencies.^16^, ^17^, ^18^^,^^31^ Two of these publications reflect how implementation of various QIPS-specific curricula benefits knowledge, though small, this growing trend of QIPS-specific curricula implementation in residency program is reassuring in preparing future pathologists for clinical practice. A lack of training in communication and error disclosure in both graduate medical education programs and within the professional pathology workforce have been identified as a major barrier in QIPS.^6^^,^^32^^,^^33^

Another barrier often cited in the literature is the necessity of shifting the culture in pathology to one that is open and accepting of diagnostic errors so that these errors can be identified, patient harm minimized or mitigated, and future errors prevented by learning from past ones.^34^, ^35^, ^36^ To attempt to shift the resident culture to one that recognizes the importance of undertaking error management in an unashamed manner, prior errors made within this program's Anatomic Pathology Department were used to model the ERCOWs. While these errors were anonymized to prevent identification of who made the error in the department, the fact that these were past errors made by faculty members was highlighted in the question sets to recognize that all pathologists, whether in training or in-practice, can learn from the mistakes of others. However, in order to do so, these errors must be recognized in a safe and accepting work environment.

Limitations to this study include the generalizability of this study to other training programs. The development of this curriculum required the input of two pathology trainees and the expertise of three pathology faculty within the program. While this expertise may not be universal at every pathology training program, numerous free resources are available to help create high-quality QIPS curricula. This includes the Institute for Healthcare Improvement Toolkits and the Association of American Medical Colleges MedEdPortal. Developing a program-specific QIPS curriculum similar to that presented here in conjunction with review of these listed resources can assist in its widespread adoption of QIPS-specific curricula in pathology training programs nationally. Additional limitations include the variable participation of trainees in different components of the curriculum, such as 57% participation rate in the lecture and SG session and 26–39% participation rate in the ERCOWs, and the lack of clinical pathology-focused topics in the curriculum.

Due to the success of this curriculum within this pathology-training program, the lecture-SG session is planned on being repeated every other year in a grand rounds lecture style. The ERCOW sets will be integrated into weekly unknown surgical pathology cases sent to trainees to challenge their diagnostic skills.

## Conclusions

This study highlights the need for QIPS-specific curriculum to be implemented within pathology training programs and the utility of a lecture, SG, and case-based curriculum in improving pathology trainee confidence and attitudes toward QIPS methodologies. This is important in both meeting ACGME milestones for pathology trainees, but more importantly in preparing a future generation of pathologists to improve and ensure patient safety.

## Funding

The article processing fee for this article was funded by an Open Access Award given by the Society of ‘67, which supports the mission of the Association for Academic Pathology to produce the next generation of outstanding investigators and educational scholars in the field of pathology. This award helps to promote the publication of high-quality original scholarship in *Academic Pathology* by authors at an early stage of academic development.

## Declaration of competing interest

The authors declare that there are no competing interests or conflicts of interest.

## References

[1] To Err Is Human (2000).

[2] Bates D.W., Singh H. (2018). Two decades since to err is human: an assessment of progress and emerging priorities in patient safety. Health Aff (Millwood).

[3] Balogh E.P., Miller B.T., Ball J.R., Improving Diagnosis in Health Care (2015). National Academies of Sciences, Engineering and Medicine.

[4] Laposata M., Cohen M.B. (2016). It's our turn: implications for pathology from the institute of medicine's report on diagnostic error. Arch Pathol Lab Med.

[5] Nakhleh R.E., Nosé V., Colasacco C. (2016). Interpretive diagnostic error reduction in surgical pathology and cytology: guideline from the college of American pathologists pathology and laboratory quality center and the association of directors of anatomic and surgical pathology. Arch Pathol Lab Med.

[6] Perkins I.U. (2016). Error disclosure in pathology and laboratory medicine: a review of the literature. AMA J Ethics.

[7] Varma M., McCluggage W.G., Shah V., Berney D.M. (2021). Pathologists can get it right the first time. J Clin Pathol.

[8] Srigley J.R., McGowan T., Maclean A. (2009). Standardized synoptic cancer pathology reporting: a population-based approach. J Surg Oncol.

[9] Becich M.J., Gilbertson J.R., Gupta D., Patel A., Grzybicki D.M., Raab S.S. (2004). Pathology and patient safety: the critical role of pathology informatics in error reduction and quality initiatives. Clin Lab Med.

[10] Nakhleh R.E., Volmar K.E. (2019). Error Reduction and Prevention in Surgical Pathology.

[11] Gallagher T.H., Mello M.M., Levinson W. (2013). Talking with patients about other clinicians' errors. N Engl J Med.

[12] Dintzis S.M., Stetsenko G.Y., Sitlani C.M., Gronowski A.M., Astion M.L., Gallagher T.H. (2011). Communicating pathology and laboratory errors. Am J Clin Pathol.

[13] Dintzis S.M., Gallagher T.H. (2009). Disclosing harmful pathology errors to patients. Am J Clin Pathol.

[14] Cohen D.A., Allen T.C. (2015). Pathologists and medical error disclosure: don't wait for an invitation. Arch Pathol Lab Med.

[15] Pathology Milestones (2019). https://www.acgme.org/globalassets/pdfs/milestones/pathologymilestones.pdf.

[16] Tucker C.M., Jaffe R., Goldberg A. (2023). Supporting a culture of patient safety: resident-Led patient safety event reviews in a pathology residency training program. Acad Pathol.

[17] Sapatnekar S., Demkowicz R., Chute D.J. (2021). Implementation of a quality and patient safety curriculum for pathology residency training. Acad Pathol.

[18] Samulski T.D., Montone K., LiVolsi V., Patel K., Baloch Z. (2016). Patient safety curriculum for anatomic pathology trainees: recommendations based on institutional experience. Adv Anat Pathol.

[19] Harris C.K., Chen Y., Yarsky B., Haspel R.L., Heher Y.K. (2022). Pathology trainees rarely report safety incidents: a review of 13,722 safety reports and a call to action. Acad Pathol.

[20] Harris C.K., Chen Y., Alston E.L. (2023). The next phase in patient safety education: towards a standardized, tools-based pathology patient safety curriculum. Acad Pathol.

[21] Thomas P. (2022).

[22] Framework for root cause analysis and corrective actions. Jt Commission. 2017; 1-18. https://www.jointcommission.org/framework_for_conducting_a_root_cause_analysis_and_action_plan/. [Accessed 20 June 2022].

[23] Whiteman K., Yaglowski J., Stephens K. (2021). Critical thinking tools for quality improvement projects. Crit Care Nurse.

[24] Rogers T.S., Wilcox R., Harm S.K. (2019). Design and implementation of a pathology-specific handoff tool for residents. Acad Pathol.

[25] Lab Management University (2022). https://store.ascp.org/productlisting/productdetail?productId=52290189.

[26] Board Review Style Questions PathologyOutlines.com. https://www.pathologyoutlines.com/review-questions.

[27] Brown A., Nidumolu A., McConnell M., Hecker K., Grierson L. (2019). Development and psychometric evaluation of an instrument to measure knowledge, skills, and attitudes towards quality improvement in health professions education: the beliefs, attitudes, skills, and confidence in quality improvement (BASiC-QI) scale. Perspect Med Educ.

[28] Malhotra N.R., Lee Y.J., Millar M.M., Cartwright P.C., Smith B.K. (2020). Experiences with quality improvement in training: a national survey of urology residents. Urology.

[29] Massagli T.L., Zumsteg J.M., Osorio M.B. (2018). Quality improvement education in residency training. Am J Phys Med Rehabil.

[30] Medbery R.L., Sellers M.M., Ko C.Y., Kelz R.R. (2014). The unmet need for a national surgical quality improvement curriculum: a systematic review. J Surg Educ.

[31] Harris C.K., Chen Y., Jensen K.C. (2022). Towards high reliability in national pathology education: evaluating the United States and Canadian Academy of Pathology educational product. Acad Pathol.

[32] Dintzis S.M., Clennon E.K., Prouty C.D., Reich L.M., Elmore J.G., Gallagher T.H. (2017). Pathologists' perspectives on disclosing harmful pathology error. Arch Pathol Lab Med.

[33] Heher Y.K., Dintzis S.M. (2018). Disclosure of harmful medical error to patients: a review with recommendations for pathologists. Adv Anat Pathol.

[34] We need to talk: pathologists, patients and diagnostic errors – part I. ThePathologist.com. 2016: 1. https://thepathologist.com/inside-the-lab/we-need-to-talk-pathologists-patients-and-diagnostic-errors-part-i. [Accessed 19 December 2023].

[35] Sirota R.L. (2005). Error and error reduction in pathology. Arch Pathol Lab Med.

[36] Nakhleh R.E. (2011). Disclosure of errors in pathology and laboratory medicine. Am J Clin Pathol.

