# Supplementary material for: Cultivating competence in error management: The development and impact of a tailored quality improvement and patient safety curriculum in pathology training
Source: Acad Pathol. 2026 Mar 19;13(2):100245. doi: 10.1016/j.acpath.2026.100245 (PMC13019064; doi:10.1016/j.acpath.2026.100245)
Supplement: Multimedia component 1 [file mmc1.pdf]

Error Management Cases of the Week

These cases are intended to highlight possible diagnostic errors in anatomic pathology.

Some of these cases have been adapted from prior errors made in the UCSF Department of Anatomic Pathology, however each has been anonymized and details have been altered to protect patient identity.

These cases are not intended to highlight prior mistakes, but rather to leverage each as an opportunity to further train future pathologists.

Learn from the mistakes of others. You can't live long enough to make them all yourself.

-Eleanor Roosevelt

*Please note that these cases are synthetic with images from different sources to simulate error management.

Instructions

- Please open the following link and input your answers in: [redacted]
  - *Remember this is not about being right or wrong – it's about learning!*
- Feel free to go as quickly or as slowly as you feel necessary.
- In two weeks time, a Powerpoint with answers and explanations will be sent out.
- If you have any questions, please direct them to [redacted]

Case #1

- History: 33 year old man with unexplained on-going diarrhea and Crohn’s disease that appears well managed. Colonoscopy identified a small ulcer in the terminal ileum and normal colon.
- You get the following random colon biopsies:

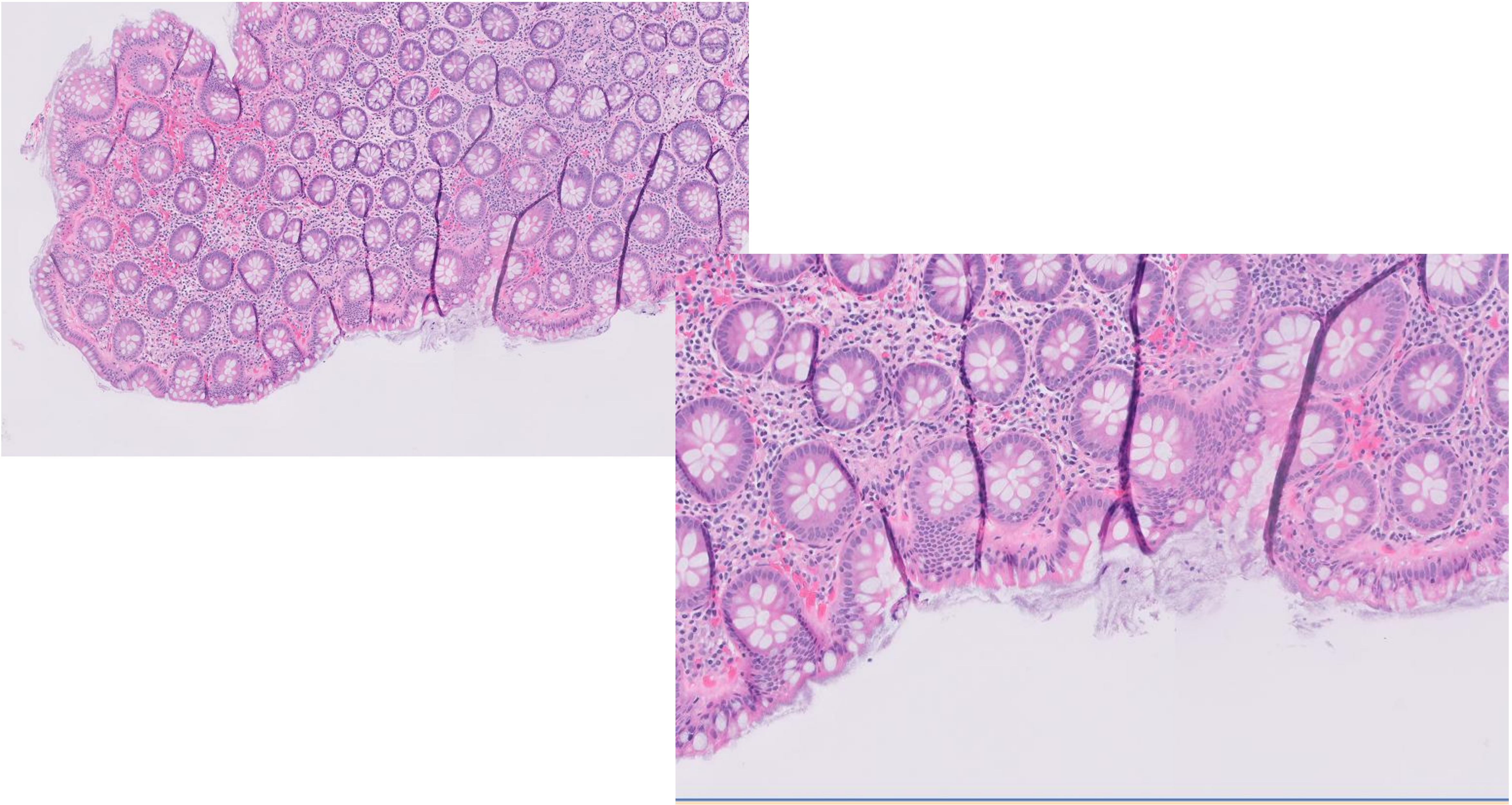

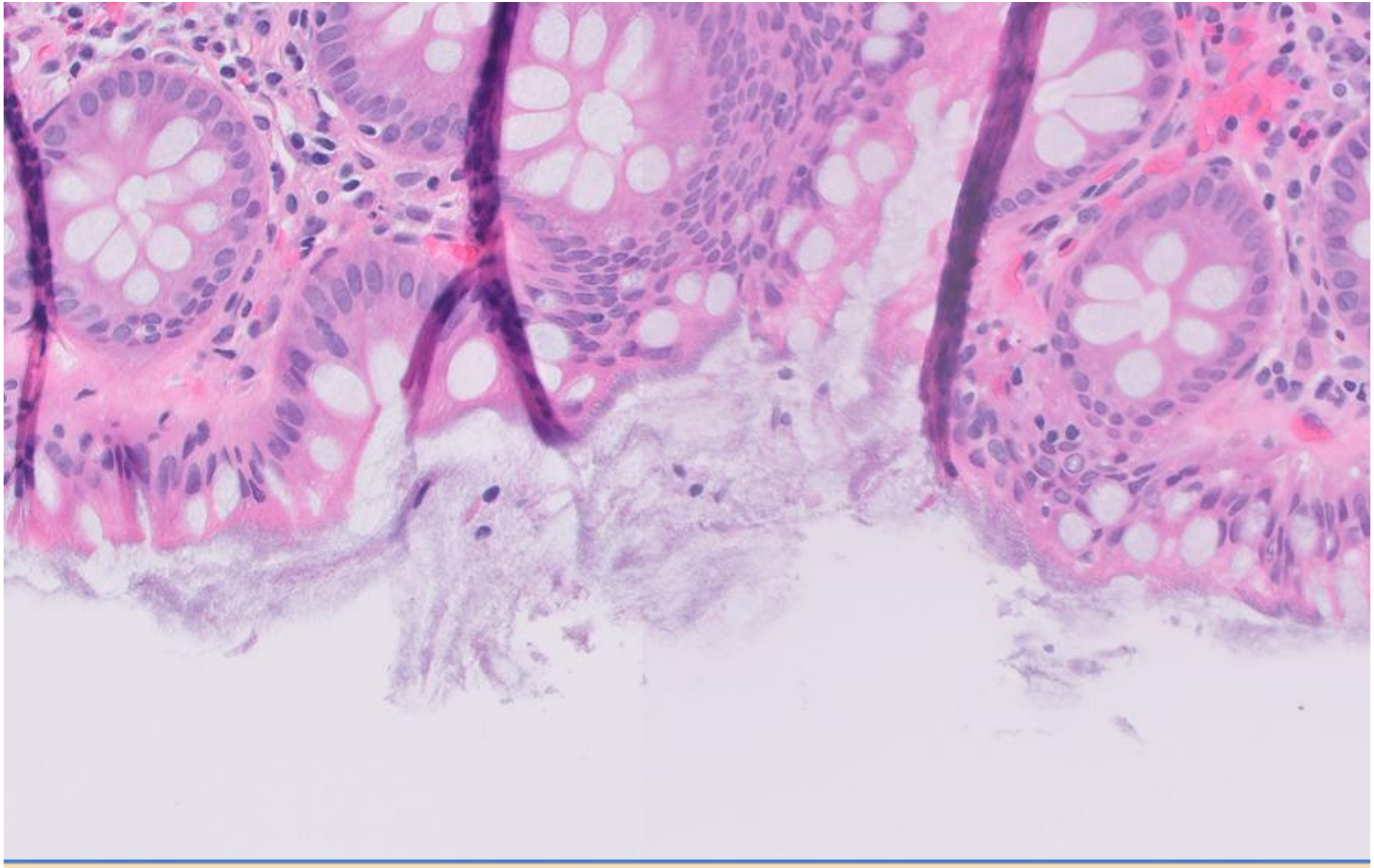

Case 1 Question 1

- What additional study would you order for this case?
  A. Steiner stain
  B. Helicobacter pylori
  C. Gram stain
  D. Treponema pallidum immunohistochemistry

You order these ancillary studies and get these results…

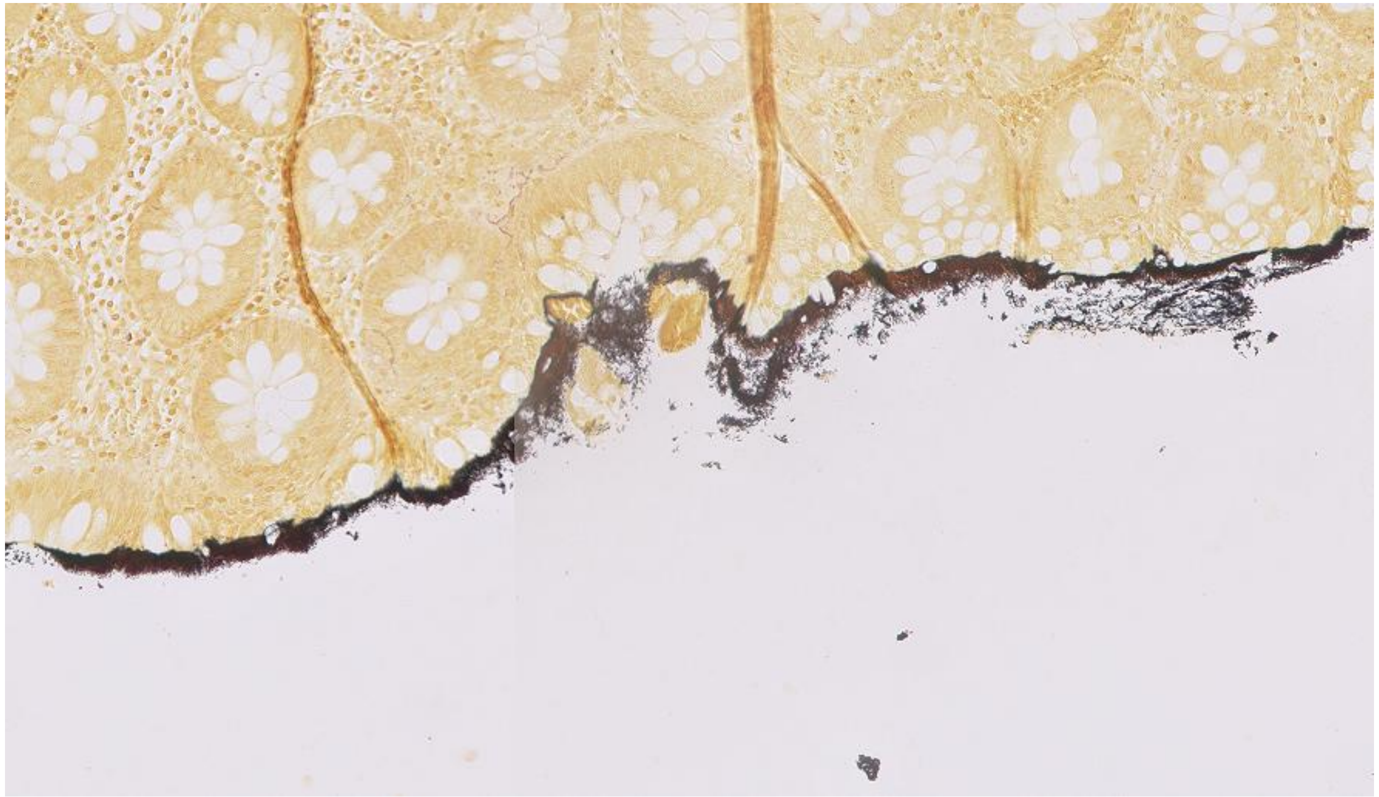

Steiner stain

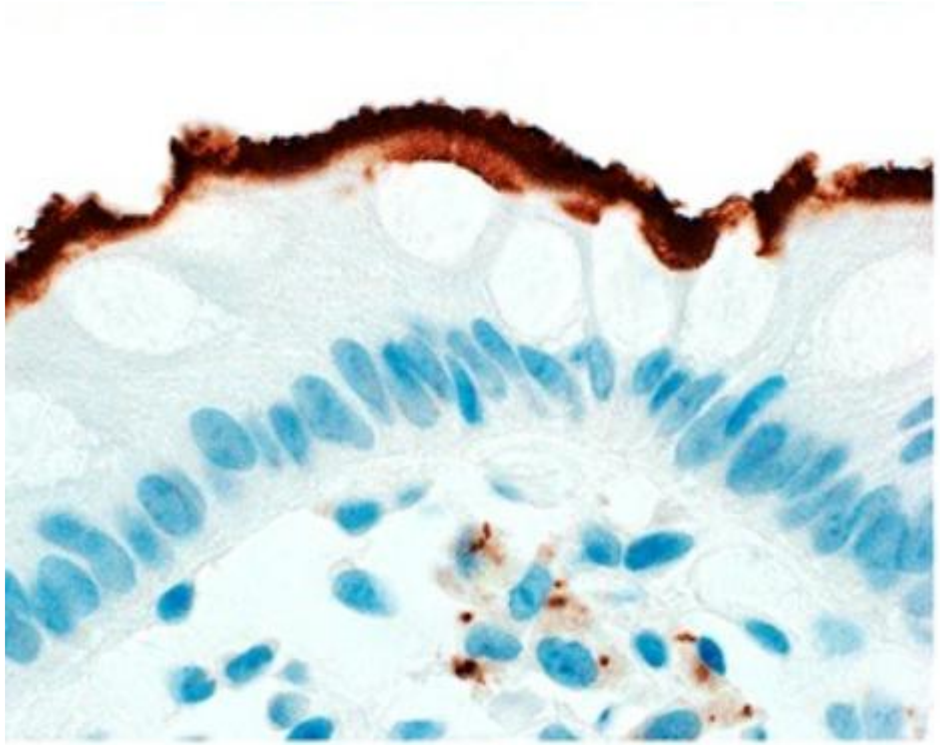

Treponema IHC

Case 1 Question 2

- What is your diagnosis?
    A. Colonic mucosa with bacterial overgrowth, correlate with endoscopy
    B. Colonic mucosa with organisms consistent with Treponema pallidum, correlate with microbiology
    C. Intestinal spirochetosis
    D. Colonic mucosa with changes suggestive of Salmonellosis

You sign out the case as…

- Colonic mucosa with organisms consistent with Treponema pallidum, correlate with microbiology and serologic studies.
- Two weeks later, the referring gastroenterologist calls you because he has performed multiple serologic tests for treponema and each is negative. Before he starts treatment, he wants to check that there was no way the specimen could have gotten mixed up?
- How do you respond? (Case 1 Question 3)
  A. Re-read your report and tell him that the Treponema IHC was positive so it must be syphilis
  B. Ignore his call, you made the correct diagnosis
  C. Review the case and consult a colleague because of the negative serologic studies
  D. Call your lab manager and ask her to see if there was any way the specimens could have been mixed up

You then decide to…

- Review the case and consult a colleague because of the negative serologic studies and call your lab manager and ask her to see if there was any way the specimens could have been mixed up
- Your colleague points you to the fact that the organisms highlighted by the IHC and Steiner stain are present only on the mucosal surface and not invasive deeper into the epithelial layer of the specimen. In this situation, the diagnosis should be intestinal spirochetosis as these organisms are not actually syphilis.

Case 1 Question 4

- What do you do next?
  - A. Amend your report
  - B. Amend your report and call the referring clinician to disclose your error
  - C. Call the referring clinician to disclose your error
  - D. Ask your colleague to call the referring clinician to disclose your error

Case 2

- You get called for a frozen called “rule out parathyroid tissue” and your resident cuts you the following frozen:

Supplemental Material 1

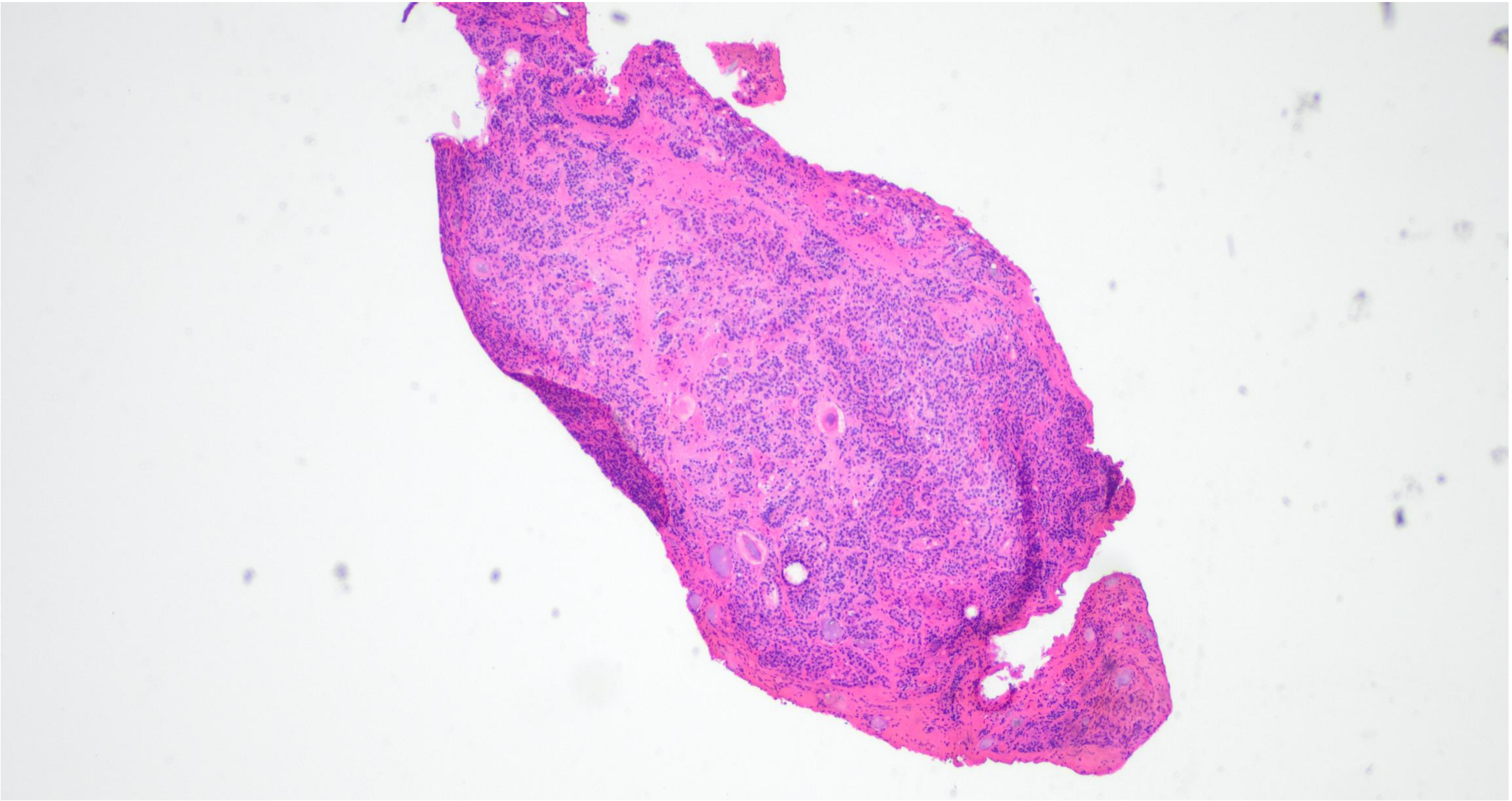

Supplemental Material 1

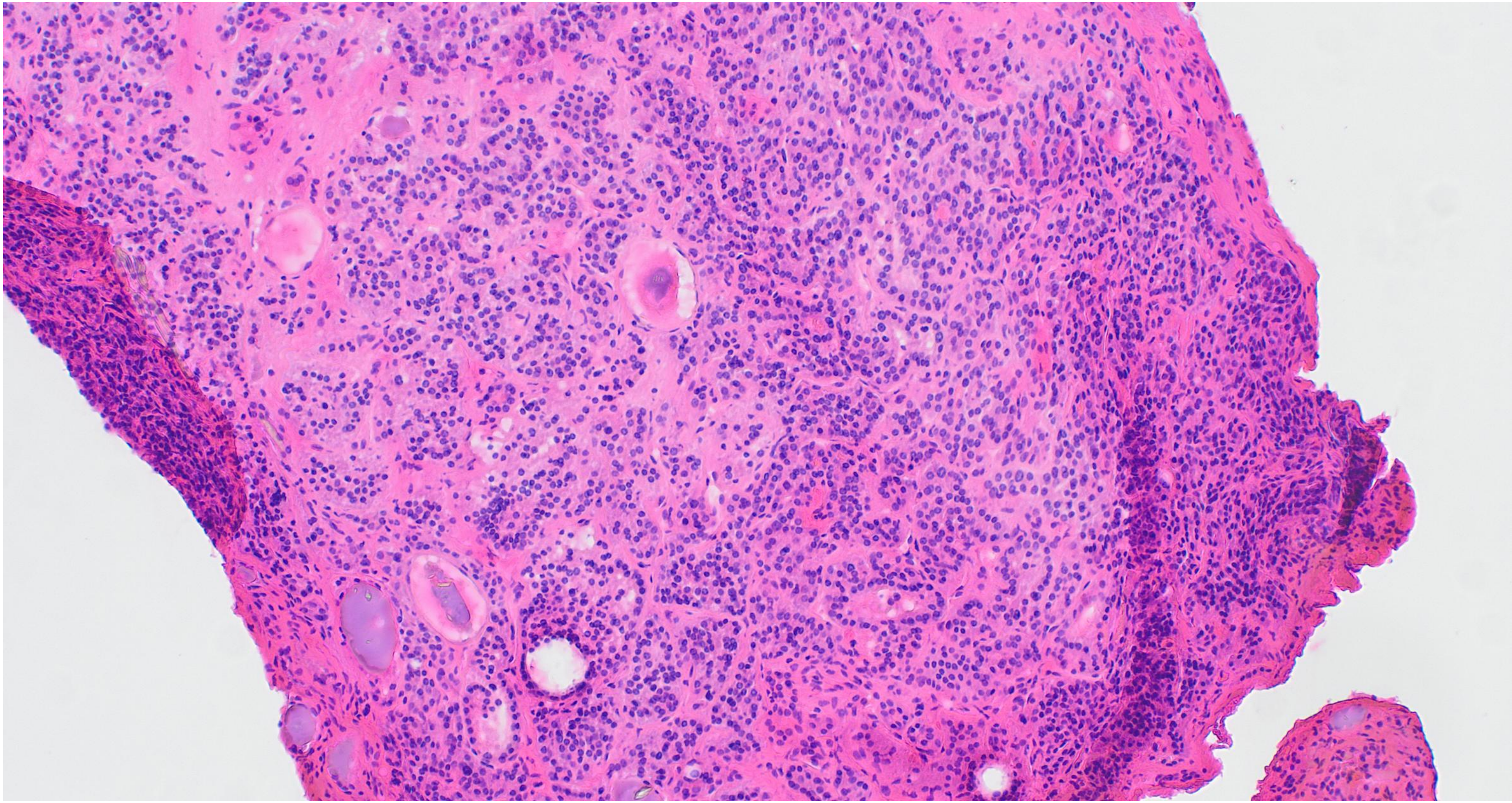

Case 2 Question 1

- What is your frozen diagnosis?
    A. Non-diagnostic, additional sampling warranted
    B. Thyroid tissue present
    C. Defer to permanents
    D. Parathyroid tissue present

You tell the surgeon that there is parathyroid tissue present…

- The surgeon says thank you and completes their procedure.
- Four days later, your colleague approaches you and asks to review this part of the case with you and shows you the following slides:

Permanent sections

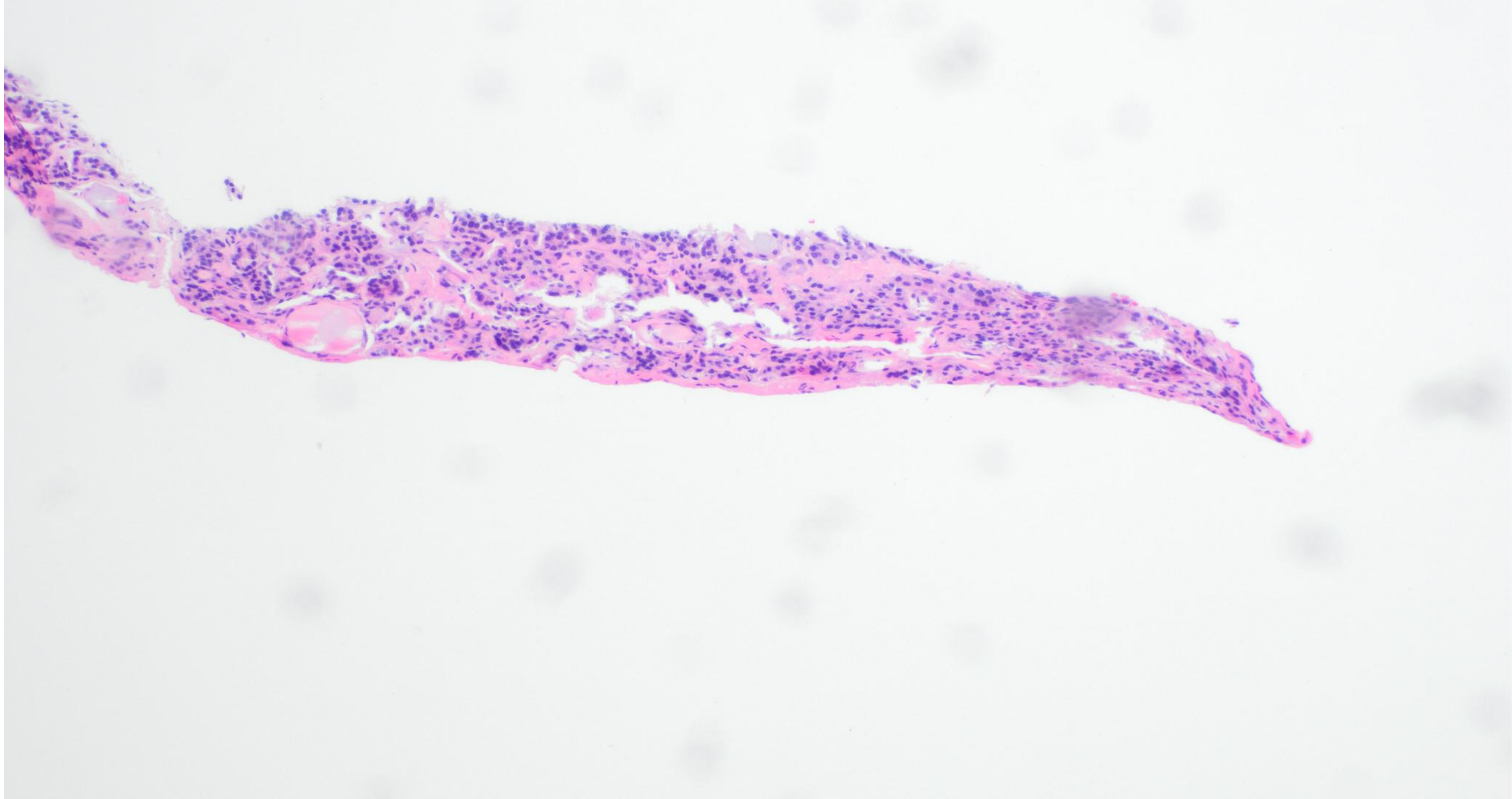

IHC stains

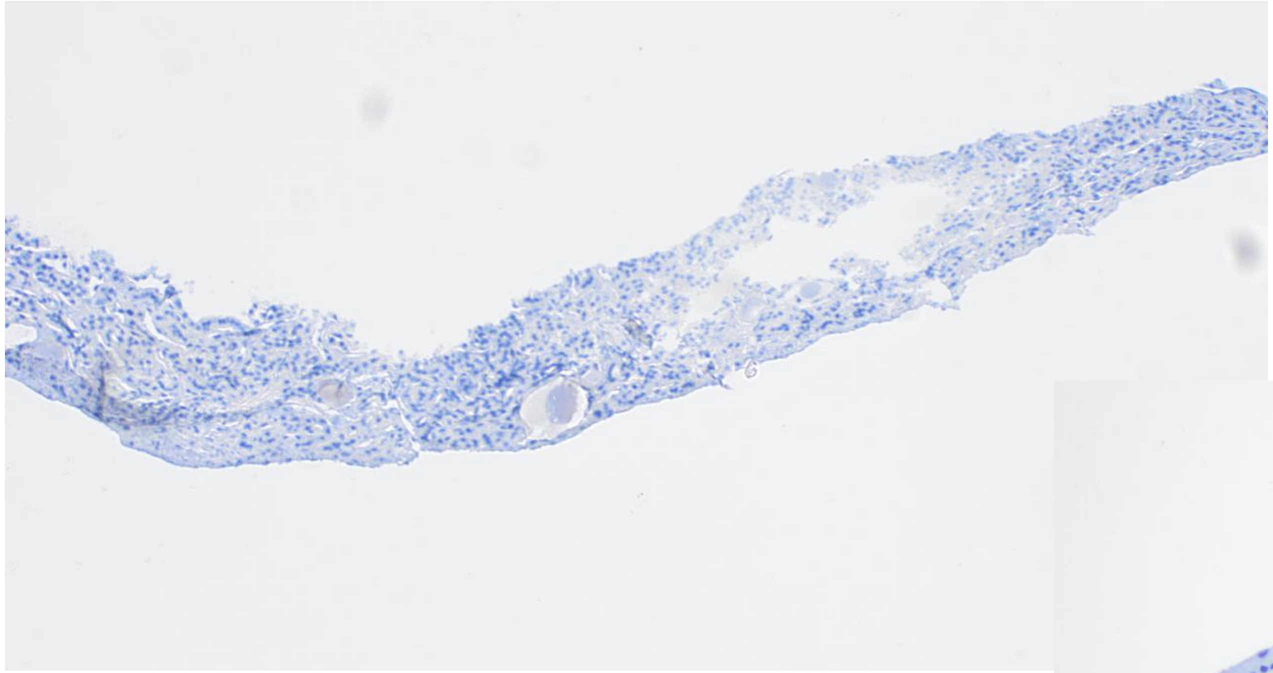

PTH

TTF1

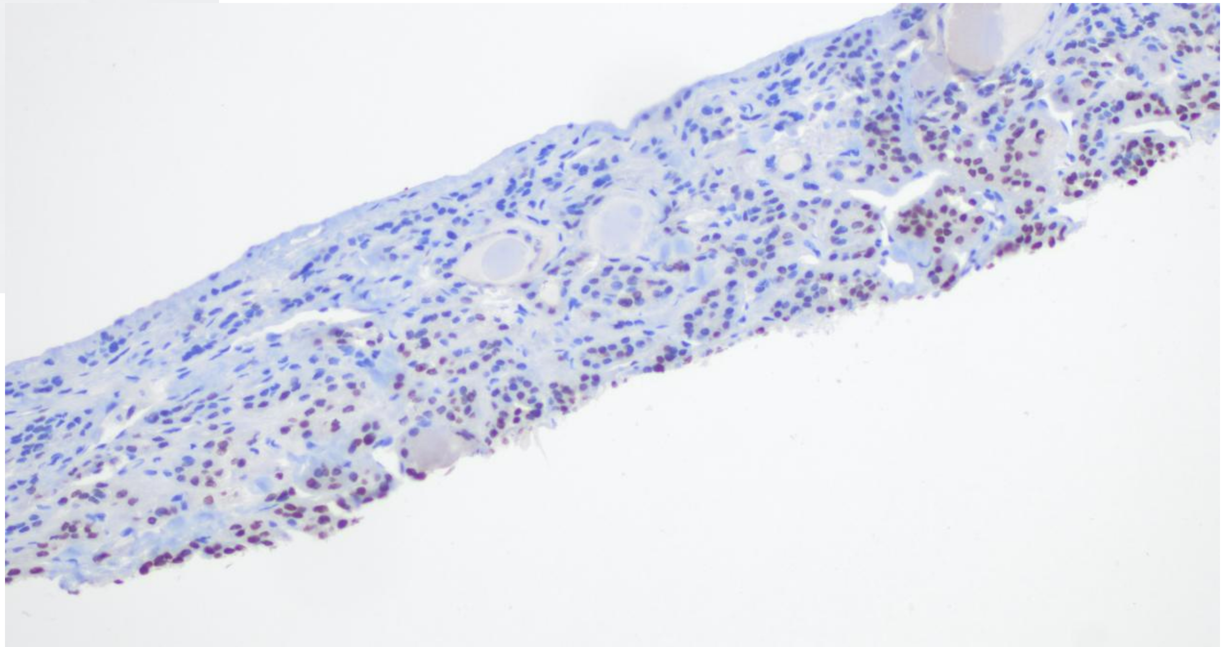

Case 2 Question 2

- What is your diagnosis now?
  A. Non-diagnostic, additional sampling warranted
  B. Thyroid tissue present
  C. Defer to permanents
  D. Parathyroid tissue present

Case 2 Question 3

- Recognizing the error you made, how should you respond to your colleague?
    A. Be angry with them. It probably wouldn’t have been a big deal if the error wasn’t caught.
    B. Blame the resident for how they cut and stained the frozen section
    C. Report, respond, review, and recover from the error
    D. Ignore the error and pretend like it didn’t happen because everyone makes mistakes

Board Style Review Question

- Emotional intelligence is based on which competencies?
  - A. Listening and communication
  - B. Personal competence and social competence
  - C. Conflict resolution and process improvement
  - D. Intrinsic personality traits

Lab Management Question

- Which of the following statements about a business plan is true?
  A. It is a narrative blueprint describing the nature of an intended business
  B. It does not include details regarding how the business is to be implemented
  C. It never exposes financial pitfalls
  D. It provides the necessary documentation for the Internal Revenue Service
  E. It is not generic but differs substantially for every industry
